# Differences in transcriptomic and metabolomic analyses of metabolites of shoots on tea plants of different ages and relevant regulatory network

**DOI:** 10.3389/fpls.2022.910895

**Published:** 2023-03-02

**Authors:** Meng Yuan Li, Yun Zhi Zhang, Zi You Zhang, Yan Hui Zhang, Qian Qian Ren, Shan Jin

**Affiliations:** ^1^Fujian Key Laboratory of Tea Science, Fujian Agriculture and Forestry University, Fuzhou, China; ^2^Yunnan National Tea Museum, Kunming, China; ^3^Yunnan Bachishan Tea Co., Ltd., Kunming, China

**Keywords:** tea plants of different ages, transcribe, metabolism, flavonoid biosynthesis, quality

## Abstract

To investigate differences in fresh leaves of tea plants at different ages in gene expression, metabolism, and dried tea quality, and to provide references to a deep exploration on metabolite differential accumulation of fresh leaves of tea plants at different ages as well as the regulation mechanism, two groups of fresh leaves from tea plants at different ages (group JP: 20-, 200-, and 1,200-year tea plants; group YX: 50-, 100-, and 400-year tea plants) were chosen as materials, and their differences in gene expression, metabolites, and metabolic regulatory network were investigated by transcriptomics and metabolomics. A total of 12,706 differentially expressed genes (DEGs) were screened from the fresh tea leaves in the JP group, of which tea-20 vs. tea-200 had the largest number of DEGs, up to 9,041 (4,459 down-regulated genes, 4,582 up-regulated genes). A total of 644 common genes in the fresh leaves of three different ages of tea plants in the JP group were differentially expressed. A total of 8,971 DEGs were screened from the fresh leaf samples of tea plants in the YX group, of which the number of DEGs obtained in the tea-50 vs. tea-400 comparison combination was the largest with a total of 3,723 (1,722 up-regulated genes and 2,001 down-regulated genes). A total of 147 common genes were differentially expressed in the fresh leaves of three different tree ages in the YX group. The pathway enrichment analysis showed that most up-regulated DEGs and their related metabolic pathways were similar in the two groups, and that the metabolic pathways of common significant enrichment included flavonoid biosynthesis, phenylpropane biosynthesis, carbon metabolism, amino acid biosynthesis, and plant pathogen interaction. The metabolomics results showed that 72 and 117 different metabolites were screened from the JP and YX groups, respectively. Most of the different metabolites in the two groups were flavonoids, phenolic acids, amino acids, and their derivatives. Among them, the number of down-regulated flavonoids in older tea plants is generally higher than the number of up-regulated flavonoids. Moreover, according to the sensory evaluation results of dried tea of fresh leaves from tea plants of different ages, tea-1200 and tea-400 showed the highest sensory evaluation scores in their groups. With increase in plant age, the fragrance of the tea was more elegant, and it changed from a dense scent to a faint scent; the tea tasted sweet and its freshness increased, while the sense of astringency was weakened and the concentration declined. Therefore, the quality difference of tea of different tree ages is mainly related to secondary metabolic pathways such as the flavonoid biosynthesis pathway. With increase in tea age, a large number of gene expression in the flavonoid biosynthesis pathway is down-regulated, which reduces the content of bitter flavonoid substances in fresh leaves and makes tea soup more mellow.

## Introduction

Plant metabolites are a source of countless medicinal compounds, while the diversity of multidimensional chemical structures has made them superior to treat serious diseases. Some have already been reported as promising alternative medicines and lead compounds for drug repurposing and discovery. These small molecules exert a wide range of effects on a plant and on other living organisms. They induce flowering, fruit set, and abscission in perennial plant growth or regulate the signal of deciduous behavior in the plant (Jiang et al., 2021; Saran et al., 2021; Solovyeva et al., 2021). Studies on tea metabolites have been a hot topic in recent years.

China is the origin of tea plants, and it is the first country in the world to discover, cultivate, and use tea plants (Yu, 1986). Tea has attracted wide attentions as a type of high-quality drink, especially unique tea plant resources and tea products like ancient tree tea and Dancong. The ancient tree tea refers to tea leaves that are prepared from fresh leaves of ancient tea plants according to some processing techniques. It generally includes ancient-tree Pu’er, ancient tree red tea, and ancient tree green tea (Yang et al., 2016). At present, some studies have demonstrated that the sensory quality of older tea plants is better than that of younger tea plants. This is mainly manifested by teas from older trees tasting more mellow and having higher resistance to brew (Liang et al., 2006). The fresh leave quality of tea plants is the basis of the high quality of a tea (Ye, 2010). Chen et al. (2011) carried out a comparative analysis on bud leaf macro-components between ancient tea plants and terraced tea plants and found that major chemical components (e.g., tea polyphenol, amino acids, and caffeine) in fresh leaves of ancient tea plants were significantly higher than those in fresh leaves of terraced tea plants. Zhou et al. (2010) studied the fragrance of terraced tea and ancient tree tea in different regions of Yunnan province and found a significant different in the fragrance of tea products. It can be seen from ancient tree tea that quality varies in different places of origin and that each product has unique characteristics (Qiao et al., 2018). At present, the research on the influence of tree age on the quality of tea mostly focuses on the quality analysis of dry tea, but there are few reports on reasons for the quality difference.

With technological development in recent years, omics research methods represented by metabolomics, transcriptomics, and proteomics have been widely used to study tea plants (Su et al., 2017). Among them, metabolomics separates and detects metabolites in the metabolism process through chromatography and mass spectrum technologies, and it can analyze metabolic pathways intuitively. Metabolomics has been extensively applied to study the influence of processing techniques, environment, and place of origin on tea quality (Dai and Lv, 2019). Based on high-throughput sequencing, transcriptomics investigates the gene expressions of a tissue or an organ from the RNA level, and it has been extensively applied to research studies concerning stress resistance and functional genes of tea plants as well as specific tea plant resources (Tang et al., 2018). Omics technology has become an important mean to study tea science (Zhao, 2019). However, multi-omics has not been used to analyze the metabolic changes in fresh leaves and formation of dry tea quality differences in wild tea plants of different ages.

In this study, fresh leaves from wild tea plants of different ages were collected as research materials, and their differences were analyzed by combining metabolomics and transcriptomics. Meanwhile, the quality difference of their dry tea was analyzed by sensory evaluation. Multiple omics approaches were used to study the differential genes, metabolites and related regulatory metabolic networks of fresh leaves of tea plants in different tree ages, and to analyze the reasons for the formation of differences in dry tea quality in different tree ages, so as to provide a reference for further exploring the differential accumulation of metabolism and its regulatory mechanism of fresh leaves of tea plants in different tree ages.

## Materials and methods

### Experimental materials

Fresh leaves of group JP were collected on 10 May 2020 from three tea plants in the same plot of n wild tea garden in Maandi town (22°40′N, 103°34′E, elevation = 920 m), Jinping Miaoyao Dai Autonomous county, Honghezhou, Yunnan province, China. The three tea plants were about 20, 200, and 1,200 years old (marked as T-20, T-200, and T-1200, respectively), and all of them are *Camellia sinensis var. Assamica*. For the location of the three tea plants in the tea garden, T-200 was about 10 m in the downhill direction of T-1200 and about 7 m in the uphill direction of T-20.

Fresh leaves of group YX were collected on 27 May 2020 from three tea plants in the same plot of the wild tea garden in Manghuai town (24°31′N, 100°24′E, elevation = 1,440 m), Yun county, Lincang city, Yunnan province, China. The three tea plants were about 50, 100, and 400 years old (labeled T-50, T-100, and T-400, respectively); all of them were *Camellia taliensis*. The three tea plants T-50, T-100, and T-400 were like the three vertices of an isosceles triangle, and the distance between two trees was about 8 m.

Tea samples of the two groups were picked with one bud and two leaves, with three biological replicates for each year. After fresh leaves were harvested, they were wrapped in tin foil paper, quickly frozen in liquid nitrogen, transferred to a refrigerator at −80°C for storage, and used for transcriptome sequencing and metabolome detection.

The age of the wild tea plants in this study is mainly determined according to the local people and the local government, combined with the appearance characteristics of the tea plants such as breast stem, crown width, and branch level. The ages of all the tested tea plants were approximate, not accurate.

### Transcriptome sequencing

#### Library construction

Agarose gel electrophoresis (AGE) and NanoPhotometer spectrophotometer were used to detect the quality and concentration of RNA samples, and Agilent 2100 bioanalyzer was used to detect the integrity of RNA samples. After the samples were tested and qualified, a library was built. The kit for library construction used the NEBNext^®^ UltraTM RNA Library Prep Kit of Illumina. The built library was diluted to 1.5 ng/μl, and the library was tested with the Agilent 2100 bioanalyzer. Later, an accurate quantization of the effective concentration of library (>2 nM) was carried out by qRT-PCR. Pooling of different libraries was performed according to machine data size under the goal, and sequencing was performed with an Illumina HiSeq platform (Conesa et al., 2016).

#### Data quality control and analysis

The image data gained with the Illumina HiSeq high-throughput sequencing platform were transformed into a lot of high-quality original data by CASAVA basic group identification. Reads with joint and N content >10% as well as low-quality (*Q* ≤ 20) reads with more than 50% basic groups were eliminated. Meanwhile, clean reads were acquired by sequencing error rate check and GC content distribution check. The indexes of reference genomes were built using HISAT2 v2.0.5. Besides, pairing terminal clean reads were compared with reference genomes^1^ using HISAT2 v2.0.5. FPKMs (fragments per kilobase of transcript per million fragments mapped) quantized the gene expression level, and reads mapped onto each gene were calculated with the feature Count. Next, the FPKMs of each gene were calculated according to the length of the gene (Liao et al., 2014).

New genes were predicted with the StringTie software (Pertea et al., 2015). The differential expression between any two samples was analyzed with the DESeq2 R software. Moreover, *P*-value was adjusted with the method proposed by Benjamini and Hochberg to control the false discovery rate (FDR), | and log2 fold change| ≥1 (Tea-20, Tea-200 and Tea-1200 inter-group screening standards were 0). Moreover, FDR <0.05 was used as the threshold to screen differentially expressed genes. A gene ontology (GO) enrichment analysis and a KEGG pathway analysis of differentially expressed genes (DEGs) were performed using clusterProfiler R.

#### Real-time fluorescence quantification PCR verification

To further test the reliability of the RNA-Seq results, a real-time fluorescence quantification PCR (qRT-PCR) verification was performed on candidate DEGs. Among DEGs gained from sequencing, a total of 11 were screened as potential genes in the biosynthesis pathway of flavonoids. All the qRT-PCR primers are designed using the Beacon Designer7.7 software, and the primer sequences are shown in Table 1.

**TABLE 1:** Primer design for qRT-PCR.

Gene	Primer sequences (forward)	Primer sequences (reverse)
b-actin	TGACCAAGCACACTCCACACTATCG	TGCCCCCTTATCATCATCCACAA
TEA024897	AGAGACTCAAGAGATGATGGATGT	TGTCAGCACTAGCAATGTCAAT
TEA027576	CAGTGGACCCTACTCTAAACC	CAGTGGACCCTACTCTAAACC
TEA008320	GAGACCTTCATCGGAGAG	AGAATCGGCAGAGCAAGTATAATC
TEA030958	GGATGCTGACAAGGACAACTAC	GCTCCATTCCAGAGGGTGTT
TEA025184	GGATGCTGACAAGGACAACTAC	ACATCGGACTCTCACCTCTC
TEA001789	CCAACTATATGCTGTCTATGTC	TCCAGGCTTGCTAACTAC
TEA006847	AATGGCATCTGATAGTTGTG	CCAGGTAGGAATCGTTCT
TEA032087	GATGAGTGATGAGGACCTTCTGA	ACCTTGGTGAGGAGTACAGTAG
TEA001157	CCACCGTCTCACCTCCTAAG	TGGCATAGTTGACCTTGTTGAATC

The above RNA was used as the template, and it was synthesized into cDNA according to the inverse transcription kit technique of a Takara Primer ScriptTM RT Reagent Kit with gDNA Eraser (Perfect Real Time). β-Actin was used as the internal reference gene. The quantitative analysis was carried out according to the specification of the TB Green™ Premix Ex Taq™ II (Tli RNaseH Plus, RR820A) kit, and the quantification PCR instrument used was BIO-RAD Cycler^®^ CFX96 Real-Time PCR System (BIO-RAD, United States). The reaction program was set at 95 C for 30 s, 95 C for 5 s, and 60 C for 30 s, for 40 cycles. The 2^–△△^CT method was used to analyze the relative changes in gene expression from real-time quantitative PCR experiments.

#### Metabolite extraction and detection

After vacuum freeze drying, the samples were ground (30 Hz, 1.5 min) into powder with a grinder (MM 400, Retsch). A total of 100 mg sample powders were collected and dissolved into 6 ml extracting solution. The mixture was put in a refrigerator (4 C) overnight (six times of swirling). After 10 min of centrifuging at the rate of 10,000 *g*, the supernatant was collected and filtered with a millipore filter (0.22 μm) and finally stored for UPLC-MS/MS analysis.

Liquid phase conditions: Waters ACQUITY UPLC HSS T3 C18 (1.8 μm, 2.1 mm × 100 mm) was used as the chromatographic column. Ultrapure water and acetonitrile were used as moving phases A and B (both were added with 04% acetic acid). The proportion of the B phase increased from 5 to 95% in the period of 0-10 min. This proportion was kept for 1 min and then decreased to 5% within 1 min. Later, it was balanced to 14 min. The flow rate, column temperature, and sample size were 35 ml/min, 40 C, and 4 μl, respectively. Mass spectral condition: temperature of the electrospray ionization, mass spectral voltage, and gas curtain were 550 C, 5,500 V, and 30 psi, respectively. The impact-induced ionization parameter was set to High. In triple quadrupole, each ion pair is scanned and tested according to optimized declustering potential and collision. The extensive targeted metabolomics test is accomplished by Wuhan Matwell Company. The data analysis of metabolites was accomplished with Analyst 1.6.3, MultiaQuant, and R. The intergroup differences of sample metabolites were simplified and maximized by PCA and orthogonal partial least square-discriminant analysis (OPLS-DA). Differential metabolites were screened by combining fold change with variable importance projection (VIP) in the OPLS-DA model. The standard was set fold change ≥2 or fold change ≤0.5 and VIP ≥1.

### Sensory evaluation analysis of groups JP and YX

#### Processing of dried tea of groups JP and YX

**FIGURE 1:**
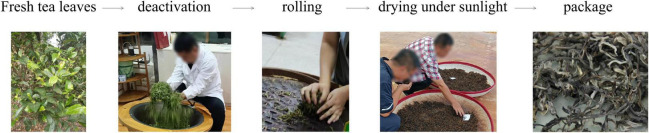
Tea processing process.

We plucked one bud and two leaves of tea plants of different ages, and obtained dried tea samples by the process of deactivation, rolling, drying under sunlight, etc., for sensory evaluation. The processing of tea is shown in Figure 1.

#### Dried tea evaluation of groups JP and YX

Sensory evaluations of dried tea samples of groups JP and YX were performed according to Standard GB/T23776-2018 (Zhang et al., 2020). By combining scoring and comments, the total score was calculated by weighted average mode. The total score was 100, including 20% of appearance, 25% of fragrance, 15% of liquor color, 30% of taste, and 10% of leave bottom. Specifically, 5 g of representative dried tea samples (tea-water ratio = 1:50) were put in corresponding evaluation cups full of boiled water. We covered the cups for 2 min. The liquor was poured into evaluation bowls quickly according to the brewing order. After evaluating liquor color, smelling the fragrance of leaf bottom, and tasting, the second brewing was performed for 5 min. The liquor was poured into bowls to evaluate its color, fragrance, taste, and leaf bottom. The color of the liquor was mainly assessed by the first brewing, while the fragrance and taste were mainly judged by the second brewing. The sensory evaluation of two groups of tea samples in different years was set up with three repetitions.

## Results and analysis

### Transcriptome analysis

#### Sequencing quality analysis

According to the transcriptome sequencing results of fresh leave samples of group JP (Tea-20, Tea-200, and Tea-1200) (Table 2), the clean data of each sample were higher than 7 Gb and the percentage of Q30 basic groups was higher than 93%. The success rate of samples matching with the reference genomes is higher than 80%, and a total of 31,540 genes were expressed. In this analysis, 12,817 new genes were identified with the StringTie software. Among them, 2,985 new genes had annotations in the GO database and 2,112 new genes were annotated onto 74 KEGG pathways. In a word, the transcriptome sequencing results were good and could provide good original data for subsequent data assembly.

**TABLE 2:** Evaluation of tea transcriptome data of group JP.

Sample	Raw-reads	Clean-reads	Clean-bases	Error rate	Q20 (%)	Q30 (%)	GC_pct (%)
Tea20-1	64,769,842	63,893,584	9.58G	0.03	97.93	94.08	43.41
Tea20-2	59,268,374	58,661,094	8.8G	0.03	97.68	93.48	43.46
Tea20-3	56,519,888	55,765,676	8.36G	0.03	97.73	93.63	43.5
Tea200-1	56,807,338	561,912,60	8.43G	0.03	97.89	94.04	44.12
Tea200-2	60,247,792	59,581,844	8.94G	0.03	97.76	93.69	43.92
Tea200-3	72,251,112	71,475,114	10.72G	0.03	97.9	94.01	44.04
Tae1200-1	55,784,798	55,147,874	8.27G	0.03	97.77	93.73	44.08
Tea1200-2	51,069,328	50,576,524	7.59G	0.03	97.73	93.65	43.88
Tae1200-3	59,290,044	58,648,896	8.8G	0.03	97.77	93.78	43.54

According to the transcriptome sequencing results of fresh leave samples of group YX (Tea-50, Tea-100, and Tea-400) (Table 3), a total of 68.37 Gb of clean data were acquired from transcriptomic sequencing, and the clean data of each sample reached 6 Gb. The percentage of Q30 basic groups was higher than 89%. The success rate of samples matching with the reference genomes was higher than 80%, and a total of 31,540 genes were expressed. In this analysis, 30,878 new genes were identified with the StringTie software. Among them, 16,329 new genes had annotations in the GO, NR, and Tremble databases. This reflects that the transcriptome sequencing results were good and could provide good original data for subsequent data assembly.

**TABLE 3:** Evaluation of tea transcriptome data of group YX.

Sample	Raw reads	Clean reads	Clean base	Error rate	Q20 (%)	Q30 (%)	GC pet (%)
Tea50-1	45,195,862	44,465,896	6.67	0.02	96.38	90.67	43.44
Tea50-2	50,450,168	48,879,880	7.33	0.02	95.94	89.83	43.69
Tea50-3	47,998,520	47,343,828	7.1	0.02	96.17	90.28	44
Tea100-1	51,610,704	50,826,614	7.62	0.02	96.09	90.06	43.89
Tea100-2	46,947802	46,223,142	6.93	0.02	96.46	90.87	44.2
Tea100-3	55,371,554	54,321,062	8.15	0.02	96.16	90.27	44.49
Tea400-1	49,572,182	48,611,350	7.29	0.02	96.23	90.33	43.11
Tea400-2	72,416,374	69,986,928	10.5	0.02	98.23	94.3	43.78
Tea400-3	45,538,182	44,619,128	6.69	0.02	96.6	91.08	43.92

#### Screening of differentially expressed genes

**FIGURE 2:**
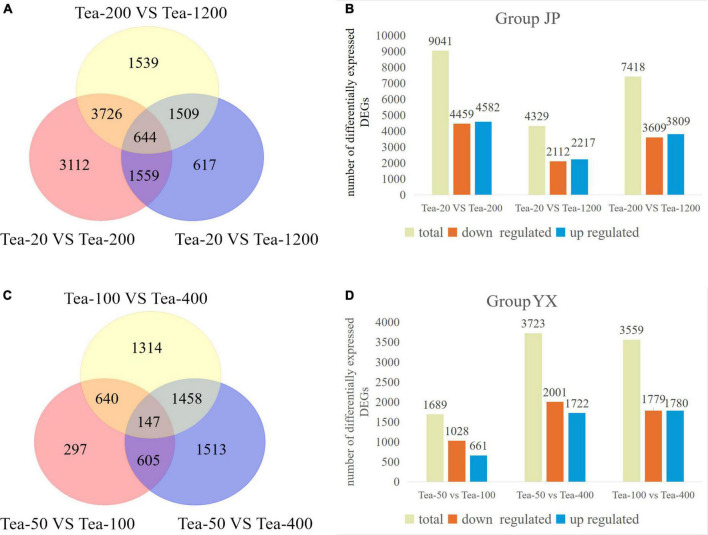
Venn diagram and column diagram of differentially expressed genes in fresh tea leaves of groups JP and YX. **(A,B)** Group JP; **(C,D)** Group YX.

Gene expression levels were quantized by feature counts, and the expression level of genes was represented by FPKMs. DEGs were screened according to | log2 (fold change)| >0 and padj <0.05 (Figure 2).

From the transcriptomic sequencing of group JP, a total of 12,706 DEGs were screened. Specifically, Tea-20 vs. Tea-200 showed the most DEGs, amounting to 9,041 (including 4,459 downregulated genes and 4,582 upregulated genes). Tea-200 vs. Tea-1200 had moderate DEGs, amounting to 7,418 (including 3,609 downregulated genes and 3,809 upregulated genes). Tea-20 vs. Tea-1200 presented the least DEGs, amounting to 4,329 (including 2,112 downregulated genes and 2,217 upregulated genes). The Venn diagram of the number of DEGs in fresh leaves of tea plants of three different ages is shown in Figure 2A. There are 644 DEGs that are differentially expressed in the fresh leaves of tea plants of three different ages.

From the transcriptomic sequencing of group YX, a total of 8,971 DEGs were screened. Tea-50 vs. Tea-400 showed the most DEGs, amounting to 3,723 (including 1,722 upregulated genes and 2,001 downregulated genes). Tea-100 vs. Tea-400 had moderate DEGs, amounting to 3,559 (including 1,780 upregulated genes and 1,779 down-regulated genes). Tea-50 vs. Tea-100 presented the least DEGs, amounting to 1,689 (including 661 up-regulated genes and 1,028 downregulated genes). The Venn diagram of the number of DEGs in fresh leaves of tea plants of three different ages is shown in Figure 2C. There are 147 DEGs in the fresh leaves of tea plants of three different ages.

Based on above analysis, groups JP and YX both presented that the number of DEGs is positively related with age gap in the comparison pair and that the number of significantly downregulated DEGs is far higher than that of significantly upregulated DEGs. This indicates that gene expression varies significantly in fresh leaves of tea plants of different ages. In other words, the number of DEGs increases with increase in tree age.

#### Gene ontology enrichment analysis

**FIGURE 3:**
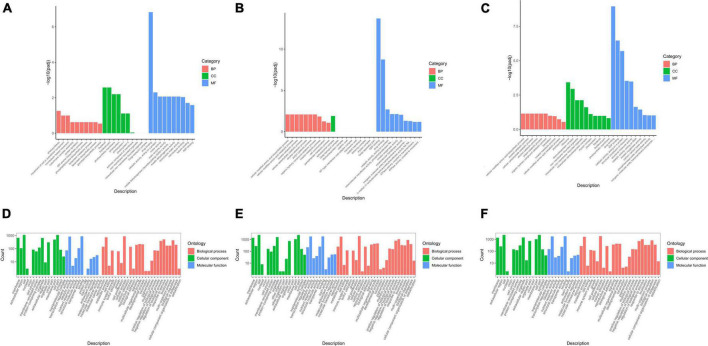
Gene ontology (GO) enrichment analysis of differentially expressed genes (DEGs) in groups JP and YX. **(A)** Tea-20 vs. Tea-200, **(B)** Tea-20 vs. Tea-1200, **(C)** Tea-200 vs. Tea-1200, **(D)** Tea-50 vs. Tea-100, **(E)** Tea-50 vs. Tea-400, and **(F)** Tea-100 vs. Tea-400.

Gene ontology is a comprehensive database that describes gene functions, and it can be divided into biological process, cellular component, and molecular function. Padj (corrected *P*-value) <0.05 was chosen as the threshold of significance analysis. The GO functionally significant enrichment is shown (Figure 3).

To understand changes in the gene expression levels of tea plants of different ages, GO analyses of groups JP and YX were carried out. It was found that among the three comparison pairs of group JP, DEGs were mostly enriched in molecular function and biological process, but that only few were enriched in cellular component. Moreover, DEGs in molecular function and biological process were significantly enriched in metabolic pathways such as photosynthesis, catalytic activity acting on proteins, ADP binding, and biosynthesis of amino acids. Therefore, gene expressions related with molecular function and biological process are significantly different among Tea-20, Tea-200, and Tea-1200. Among the three comparison pairs of group YX, DEGs of Tea-400 vs. Tea-50 and Tea-400 vs. Tea-100 were mainly enriched in cellular composition, molecular function, and biological process. Metabolic pathways with significant enrichment of DEGs include membrane and organelle, catalytic activity, and transfer activity as well as cell process, metabolic process, and biological regulation.

#### KEGG enrichment analysis of differentially expressed genes

**FIGURE 4:**
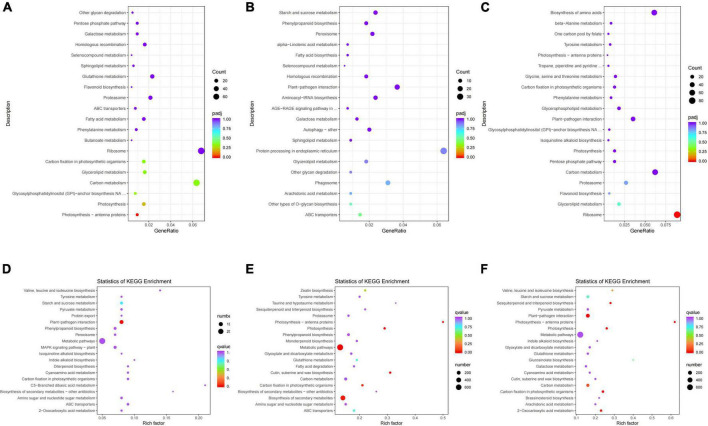
KEGG pathway enrichment analysis of differentially expressed genes (DEGs) in panels **(A–C)** group JP and **(D–F)** group XY. **(A)** Tea-20 vs. Tea-200, **(B)** Tea-20 vs. Tea-1200, **(C)** Tea-200 vs. Tea-1200, **(D)** Tea-50 vs. Tea-100, **(E)** Tea-50 vs. Tea-400, and **(F)** Tea-100 vs. Tea-400.

To further study the differences between groups JP and YX on the molecular level, KEGG pathway enrichment was carried out on DEGs. The first 20 KEGG pathways with DEG enrichment were selected (Figure 4).

According to the KEGG enrichment analysis results of group JP, DEGs of Tea-20 vs. Tea-200 (Figure 4A) were enriched significantly in 110 KEGG pathways, including the photosynthesis pathway, glycerolipid metabolic pathway, carbon metabolism and amino acids, biosynthesis of flavonoids, biosynthesis of phenylalanine metabolism, and so on. DEGs of Tea-20 vs. Tea-1200 (Figure 4B) are mainly annotated in 100 KEGG pathways. Among them, KEGG paths with significant enrichment of DEGs include endoplasmic reticulum protein processing, carbon metabolism, biosynthesis of amino acids, and biosynthesis of phenylpropane. There are 934 DEGs of Tea-200 vs. Tea-1200 (Figure 4C) that are related with 108 pathways such as ribosome, carbon metabolism, biosynthesis of amino acids, glycerolipid metabolism, biosynthesis of flavonoids, and plant pathogen interaction.

According to the KEGG enrichment analysis results of group YX, 638 DEGs of Tea-50 vs. Tea-100 (Figure 4D) were annotated onto 126 metabolic pathways. Pathways with significant DEG enrichment include the metabolism pathway, biosynthesis of phenylpropane, plant pathogen interaction, biosynthesis of secondary metabolites, biosynthesis of flavonoidsm, etc. For Tea-50 vs. Tea-400 (Figure 4E), 1,494 DEGs were annotated by 135 KEGG pathways, which were mainly related with metabolism and genetic information processing. Significant enrichments occur in metabolic pathways, biosynthesis of secondary metabolites, and biosynthesis of flavonoids. For Tea-100 vs. Tea-400 (Figure 4F), 1,462 DEGs were related with 134 metabolic pathways, and significant enrichment occurs in plant pathogen interaction, metabolic pathway, and carbon metabolism.

To sum up, among the three comparison pairs of Tea-20, Tea-200, and Tea-1200, significant enrichment of DEGs was observed in carbon metabolism and biosynthesis of amino acids. Among the three comparison pairs of Tea-50, Tea-100, and Tea-400, significant enrichment of DEGs was observed in metabolic pathway, biosynthesis of secondary metabolites, plant pathogen interaction, biosynthesis of flavonoids, and biosynthesis of phenylpropane. Among the six comparison pairs, significant enrichment of DEGs was observed in biosynthesis of flavonoids and biosynthesis of phenylpropane.

#### Analysis of related pathways of flavonoid synthesis

**FIGURE 5:**
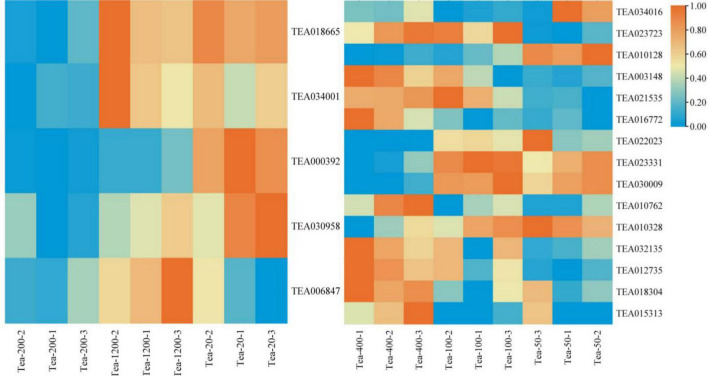
Heat map of the flavonoid synthesis pathway and related gene expression in tea shoots of different years. The left heat map for Group JP, and the right heat map for Group YX.

Flavonoids are the most important secondary metabolites of tea plants and play a decisive role in tea flavor and healthcare function. The flavonoid biosynthesis pathway starts from the phenylpropane metabolism pathway. L-Phenylalanine in the starting substrate in the pathway; the pathway of phenylpropane generates coumaroyl A and trans-coumaryl coenzyme A, which are precursor substances of the synthesis pathway of flavonoids. The results of groups JP and YX groups are as follows (Figure 5):

The expression levels of *CHS* (TEA018665), *C4H* (TEA034001), and *DoOMT* (TEA030958) in Group JP of tea plants did not follow certain rules with the change of tea plants age, that is, the expression of *CHS*, *C4H*, and *DoOMT* were higher in Tea-20 and Tea-1200 than that in Tea-400. However, the expression of *F3H* (TEA006847) in Group JP of tea plants gradually decreased with the decline of the tea plants age, that is, the expression of *F3H* was the highest in Tea-1200, the expression of Tea-20 was the lowest. The expression level of *CCoAOMT* (TEA000392) was the highest in Tea-20, the expression of Tea-200 was the lowest. In group YX, the expression levels of *FLS* (TEA023723, TEA010762), *HCT* (TEA003148, TEA032135), *LAR* (TEA021535), *C4H* (TEA016772), and *COMT* (TEA012735) showed an increasing trend with increase in tea plant years; that is, the expression levels of these genes were highest in Tea-400 tea plants, the expression levels of Tea-50 were the lowest. However, the expression levels of *CHS* (TEA023331), *DFR* (TEA030009), and *FLS* (TEA010328) in Group YX of tea plants were significantly higher in Tea-50 and Tea-100, and the expression of *HCT* (TEA010128) was also highest in Tea-50.

#### Pathway analysis of phenylpropane biosynthesis

**FIGURE 6:**
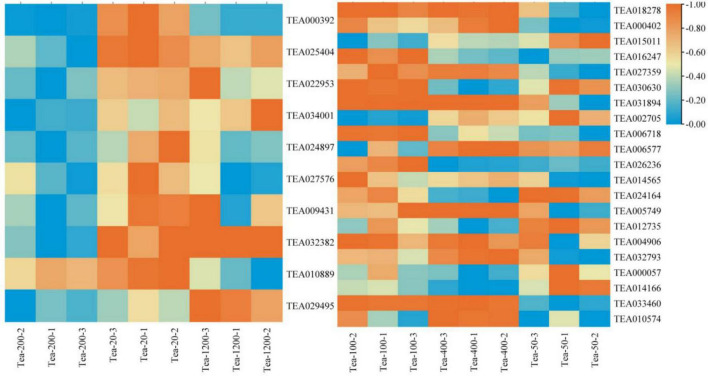
Heat map of the phenylpropane biosynthesis pathway and related gene expression in tea shoots of different years. The left heat map for Group JP, and the right heat map for Group YX.

Synthesis of polyphenol in tea plants involves the pathway of shikimic acid, pathway of phenylpropane, and pathway of flavonoids (Han, 2018). The differential gene analysis of the phenylpropane biosynthesis pathway between Groups JP and XY group found that (Figure 6):

In the Group JP, the expression levels of *CCoAOMT* (TEA000392), *CAD* (TEA024897), *POD* (TEA027576) in Tea-20 were significantly higher than that in Tea-200 and Tea-1200; while the expression of anthocyanidin 3-O-glucosyltransferase5 (TEA029495) was the highest in Tea-1200. The expression levels of CAD (TEA025404), *POD6* (TEA022953), *C4H* (TEA034001), *4CL* (TEA009431), and *CYP736A* (TEA032382) in Tea-20 and Tea-1200 were the significantly higher than that in Tea-200. The expression of *REF1* (TEA010889) gradually increased with the decrease of tea plant years. In Group YX, the expression levels of *POD11* (TEA024164), *F5H* (TEA000057), and *PAL* (TEA014166) increased with the age of tea plants gradually decrease; while *CAD* (TEA018278, TEA000402), *POD4* (TEA027359), *POD5* (TEA005749), *CCR* (TEA032793), and *COMT* (TEA010574) gradually increased with the increase of tea plant years, and the expression levels of *POD2* (TEA031894) and *REF1* (TEA033460) in Tea-400 and Tea-100 were significantly higher than that in Tea-50.

#### qRT-PCR verification of transcriptomic results

**FIGURE 7:**
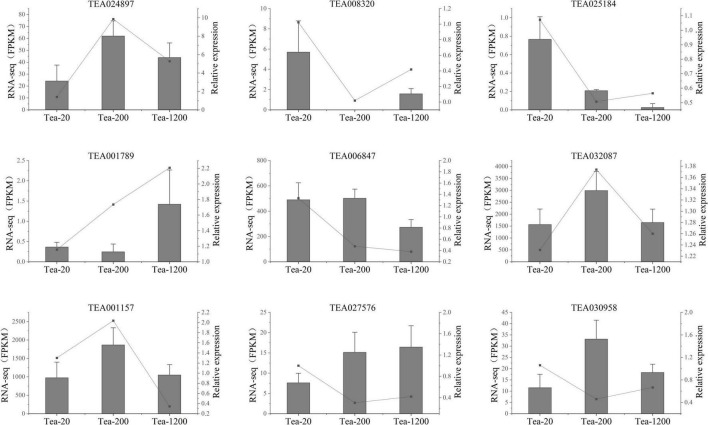
qRT-PCR verification of transcriptomic sequencing. Fragments per kilobase of transcript per million fragments mapped (FPKM) of transcriptome data (gray columns) and relative expressions of qRT-PCR test (black points) are represented.

To further verify the transcriptomic sequencing data, nine candidate genes were chosen for qRT-PCR test, including biosynthesis pathway of phenylpropanoids, biosynthesis pathway of flavonoids, and genes related with photosynthesis. According to the results, the expression mode of the selected genes is basically consistent with the transcriptomic sequencing results, indicating reliability and repeatability of the RNA-seq data (Figure 7). Therefore, the receipts obtained in this study can be used to study the differences in metabolites of tea shoots of different years and their regulatory mechanisms.

### Metabonmics analysis

#### Differential metabolite statistics

**FIGURE 8:**
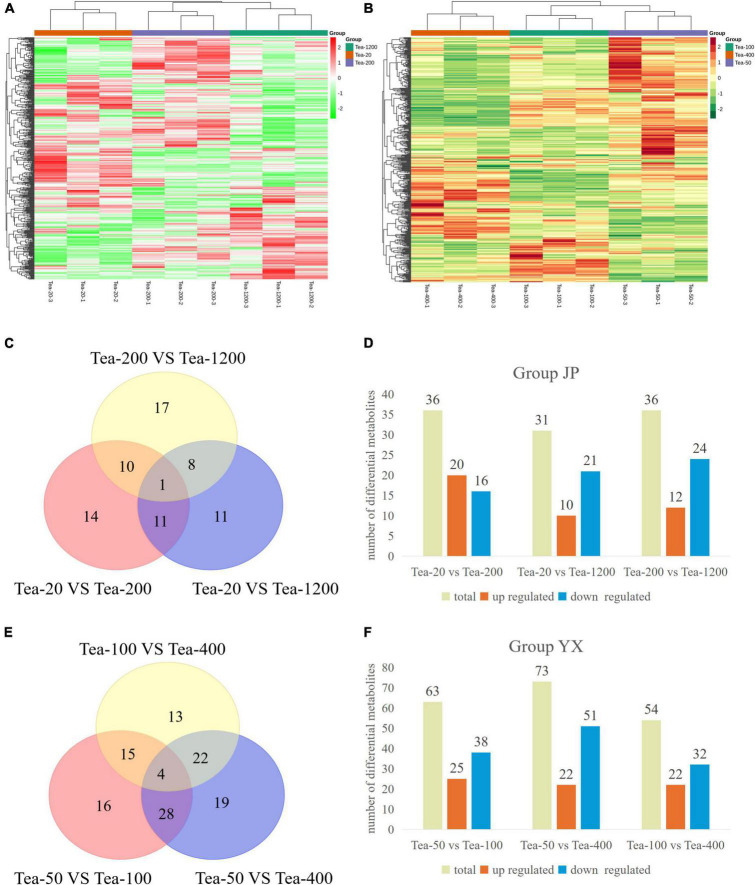
**(A,B)** Cluster heat map and **(C–F)** statistical map of differential metabolites of fresh leaves of trees of different ages.

By comparing the metabolome database built by Maiwei company, 433 and 440 metabolites were tested in Groups JP and YX, mainly including flavonoids, lipids, amino acids and derivatives, nucleotides and derivatives, organic acids, and so on. Moreover, it can be found from the heat map clustering analysis based on concentration data of metabolites that all biological repetitions cluster well. This reflects that the metabolomics data are reliable (Figures 8A,B).

The metabolomics results of group JP (Figures 8C,D) showed that a total of 72 differential metabolites were screened. Thirty-six differential metabolites were screened from Tea-20 vs. Tea-200, including 20 significantly upregulated differential metabolites and 16 significantly downregulated differential metabolites. Among them, the significantly upregulated differential metabolites mainly include flavonoids such as vitexin, pufferin, citronellin, and naringenin, and the significantly downregulated differential metabolites mainly include procyanidin, catechin, 3-O-p-coumaroyl Quinic acid, etc. Similarly, 36 differential metabolites were screened from Tea-200 vs. Tea-1200, including 12 significantly upregulated differential metabolites and 24 significantly downregulated differential metabolites. Among them, the significantly upregulated differential metabolites mainly include flavonoids such as myricetin, proanthocyanidin, and (−)-epiafzelechin, and the significantly downregulated differential metabolites mainly include vitexin, mannitol, and other substances. However, Tea-20 vs. Tea-1200 had the lowest number of differential metabolites with a total of 31 (10 significantly up-regulated and 21 significantly down-regulated), and the differential metabolites were mainly flavonoids and phenolic acids. Compared with Tea-20 and Tea-200, the number of down-regulated metabolites in the fresh leaves of Tea-1200 was more than the number of the upregulated, and most of the down-regulated metabolites were flavonoids. This indicates that the content of most flavonoids is lower in the fresh leaves of the older tea plants than in those of the younger tea plants.

Similar phenomena were discovered in group XY. A total of 117 differential metabolites were screened in group XY (Figures 8E,F). Tea-50 vs. Tea-100 had 63 differential metabolites (25 up-regulated and 38 down-regulated). Among them, most of the differential metabolites are flavonoids and phenolic acids such as tangerine peelin, vitexin, isovitexin, coniferoside, dregnoside, and luteolin. In Tea-50 vs. Tea-400, there were 73 differential metabolites, including 22 significantly up-regulated ones and 51 significantly downregulated ones. Most of the differential metabolites are flavonoids such as myricetin, vitexin, and citronella. In Tea-100 vs. Tea-400, there were 54 differential metabolites, including 22 upregulated ones and 32 significantly downregulated ones. Most of which were still flavonoids and phenolic acids.

It can be seen that the different metabolites of fresh tea leaves in groups JP and YX at different years are mainly flavonoids, tannins, and phenolic acids. Compared with the younger tea plants, the number of downregulated flavonoids in the older tea plants is generally more than the number of upregulated flavonoids, which may be related to the gradual aging of the older tea plants.

#### Analysis of differential metabolite content

**FIGURE 9:**
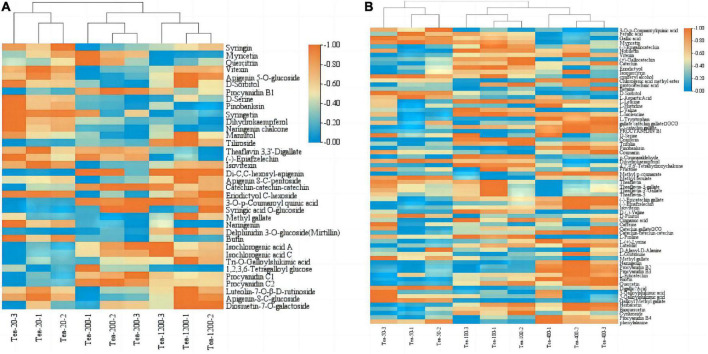
Expression analysis of different metabolites in fresh leaves of tea plants of different tree ages in groups JP **(A)** and YX **(B)**.

By differential metabolite content analysis of fresh tea leaves of groups JP and YX at different years, some differential metabolites show a regular change with increase in tree age, as shown in Figure 9.

Among the 72 differential metabolites in group JP, the contents of amino acids and their derivatives, such as D-serine, and most flavonoids, such as butin, naringenin, vitexin, pinobanksin, syringen, and dihydrokaempferol, gradually decreased with increase in tea plant age. However, the content of proanthocyanidins such as catechin, procyanidin B1, C1, and C2, and phenolic acids such as 3-o-p-coumaroyl quinic acid, methyl gallate, isochlorogenic acid A, and isochlorogenic acid C increased significantly with increase in tea age. The contents of myricetin and quercitirin in Tea-200 were highest, while those in Tea-1200 were lowest; The contents of D-sorbitol, vitexin. and apigenin 5-o-glucoside were lowest in Tea-200 and highest in Tea-20.

Among the 117 differential metabolites in group YX, the contents of flavonoids such as gallic acid, myricetin, protocatechuic acid, and dihydrokaempferol, phenolic acids such as ferulic acid, coniferyl alcohol, and chlorogenic acid methyl ester, and amino acids and their derivatives such as L-aspartic acid, L-histidine, L-leucine, and L-isoleucine decreased with increase in tea age. However, the contents of flavonoids such as gallate catechin gallate (GCG), methyl gallate, naringenin, L-epicatechin, and quercetin, phenolic acids such as methyl p-coumarate, and procyanidins B2, C1, and C2 gradually increased with increase in tea age. The content of metabolites such as flavonoids (−)–epigalactocatechin, (+)–galactocatechin, nobiletin, vitexin, and eriodictyol was highest in Tea-100 and lowest in Tea-50 trees.

#### KEGG enrichment analysis of differential metabolites

**FIGURE 10:**
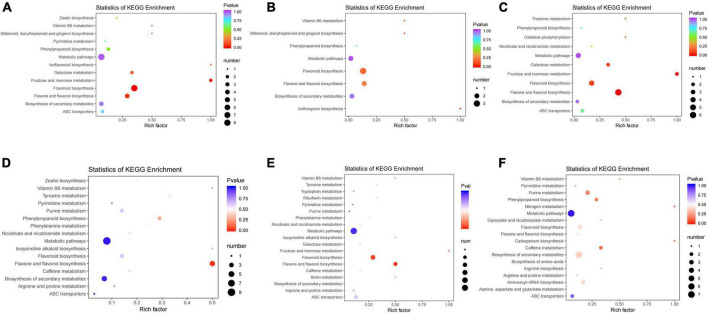
KEGG pathway enrichment analysis of differential metabolites of groups JP and YX. **(A)** Tea-20 vs. Tea-200, **(B)** Tea-20 vs. Tea-1200, **(C)** Tea-200 vs. Tea-1200, **(D)** Tea-50 vs. Tea-100, **(E)** Tea-50 vs. Tea-400, and **(F)** Tea-100 vs. Tea-400.

The KEGG database is the integrated metabolic pathway inquiry provided for researchers, and it is an important tool for metabolic network analysis of organisms. KEGG annotation and enrichment analysis were performed on the screened differential metabolites (Figure 10).

According to the KEGG analysis of differential metabolites in group JP, differential metabolites of Tea-20 vs. Tea-200 were mainly enriched in 44 metabolic pathways. Metabolic pathways with significant enrichment mainly include biosynthesis of flavonoids, biosynthesis of flavone and flavonol, and biosynthesis of flavones as well as metabolism of galactose, fructose, mannose, and vitamin B6. Differential metabolites of Tea-20 vs. Tea-1200 were mainly enriched in 13 metabolic pathways, and metabolic pathways of significant enrichment involve biosynthesis of anthocyanin, biosynthesis of flavonoids, flavone, and flavonol as well as metabolism of vitamin B6. Differential metabolites of Tea-200 vs. Tea-1200 were mainly enriched in 26 metabolic pathways, and metabolic pathways of significant enrichment involve biosynthesis of flavonoids, flavone and flavonol, metabolism of galactose, fructose, and mannose, oxidative phosphorylation, and thiamine metabolism. Therefore, metabolic pathways with common enrichment of differential metabolites among the three comparison pairs of group JP mainly involve biosynthesis of flavonoids, flavone, and flavonol as well as metabolism of galactose, fructose, mannose, and vitamin B6.

According to the KEGG analysis of differential metabolites of group XY, differential metabolites of Tea-50 vs. Tea-100 were mainly enriched in 33 metabolic pathways. Significant enrichment pathways involve biosynthesis of flavonoids, flavone, flavonol, and phenylpropane as well as metabolism of tyrosine and vitamin B6. Differential metabolites of Tea-50 vs. Tea-400 were mainly enriched in 48 metabolic pathways. Metabolic pathways of significant enrichment involve biosynthesis of flavonoids, flavone, and flavonol as well as metabolism of fructose, mannose, phenylalanine, tyrosine, and vitamin B6. Differential metabolites of Tea-50 vs.Tea-100 were mainly enriched in 37 metabolic pathways. Metabolic pathways of significant enrichment involve metabolism of caffeine and purine as well as biosynthesis of phenylpropane, secondary metabolites, carbapenems, arginine, flavonoids, flavone, and flavonol. Therefore, metabolic pathways of common significant enrichment of differential metabolites in three comparison pairs of group XY involve biosynthesis of flavone, flavonol, and phenylpropane as well as metabolism of vitamin B6, fructose, and mannose.

To sum up, metabolic pathways of common significant enrichment of differential metabolites of groups JP and XY mainly involve biosynthesis of flavonoids, flavone, flavonol, and phenylpropane as well as metabolism of fructose and mannose.

#### Differential metabolite expression analysis

By the metabolome analysis of fresh tea leaves in groups JP and YX in different years, it was found that most of the differential metabolites of fresh tea leaves in different years were flavonoids and phenolic acids, and the results are as follows (Figure 9).

A total of 433 metabolites were detected in fresh tea leaves of group JP. Among them, the content of amino acids and their derivatives D-serine, naringenin, vitexin, butin, pinobanksin, syringetin, and dihydrokaempferol, and most of the flavonoids gradually decreased with increase in tea plant years. Catechin-catechin-catechin, procyanidin B1, procyanidin C1, procyanidin C2, and other proanthocyanidins, and phenolic acids such as 3-O-p-coumaroyl quinic acid, isochlorogenic acid A, isochlorogenic acid C, and other phenolic acids, with increase in tea plant years, their expression levels increased significantly in the Tea-200 and Tea-1200 tea plants. The expression levels of myricetin, quercitrin, theaflavin 3,3′-digallate, (−)-epiafzelechin, and 1,2,3,6-tetragalloyl glucose are the lowest in the tea plant of Tea-1200. The expression levels of D-sorbitol, apigenin 5-O-glucoside, mannitol, isovitexin, apigenin 8-C-pentoside, eriodictyol C-hexoside, methyl gallate, luteolin-7-O-β-D-rutinoside, and apigenin-8-C-glucoside are the lowest in the tea plant of Tea-200.

A total of 440 metabolites were detected in fresh tea leaves of group XY; among them were flavonoids such as gallic acid, myricetin, protocatechuic acid, and dihydrokaempferol, phenolic acids such as ferulic acid, coniferyl alcohol, chlorogenic acid methyl ester, and amino acids such as L-aspartic acid, L-histidine, L-leucine, and L-isoleucine. The contents of derivative substances all showed the characteristics of gradually decreasing with increase in tea plant years. Flavonoids such as GCG, methyl gallate, naringenin, L-epicatechin, quercetin, phenolic acids such as methyl p-coumarate and the content of procyanidin B2, procyanidin C1, procyanidin C2 increased gradually with increase in tea plant years. The expression levels of flavonoids (−)-epigallocatechin, (+)-gallocatechin, nobiletin, vitexin, eriodictyol, and other metabolites in Tea-100 tea plant is highest, and its expression level is lowest in Tea-50 tea plants.

#### Metabolomics and transcriptomics conjoint analysis

**FIGURE 11:**
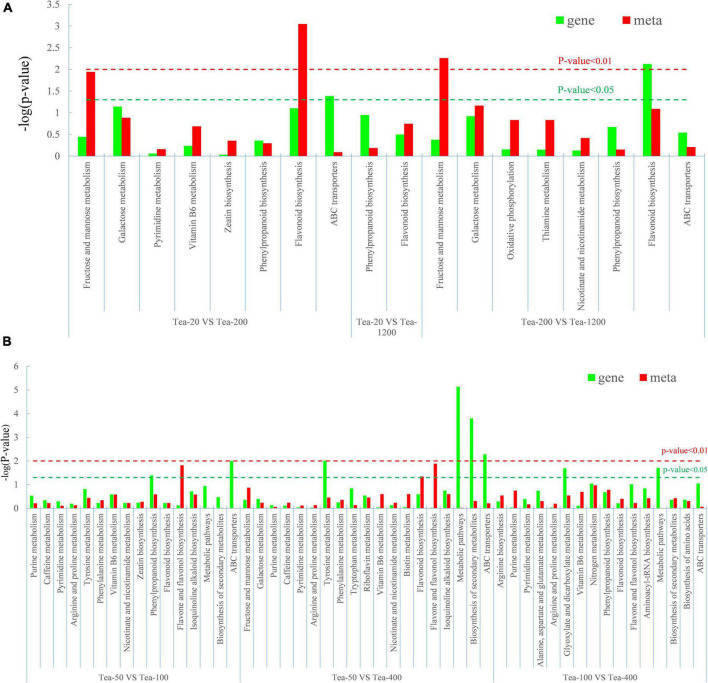
Degree of relevant pathway enrichment of differential metabolites and differentially expressed genes (DEGs). **(A)** Group JP and **(B)** Group YX.

The results of the KEGG enrichment analysis of the differential genes and differential metabolites of fresh tea leaves in groups JP and XY showed that (Figure 11):

Differential metabolites of Tea-20 vs. Tea-200 have the strongest enrichment in the biosynthesis pathway of flavonoids, and that differential metabolites of Tea-200 vs. Tea-1200 have relatively stronger enrichment in metabolic pathways of fructose and mannose. Differential metabolites were mainly enriched in biosynthesis pathways of flavonoids. In group YX, differential metabolites of Tea-50 vs. Tea-100 have strong enrichment in biosynthesis pathways of flavone and flavonol, while DEGs were enriched in biosynthesis pathways of phenylpropane. In Tea-50 vs. Tea-400, DEGs have a relatively high enrichment degree in the biosynthesis pathway of metabolic pathway and secondary metabolites. This demonstrates that the fresh leaves of tea plants of different ages differ significantly in metabolic pathways and synthesis of secondary metabolites, especially in the biosynthesis pathway of flavonoids and metabolic pathways of fructose and mannose. Gene expression and metabolite synthesis are significantly different among the tea plants of different ages.

#### Differential gene and differential metabolite correlation network analysis

**FIGURE 12:**
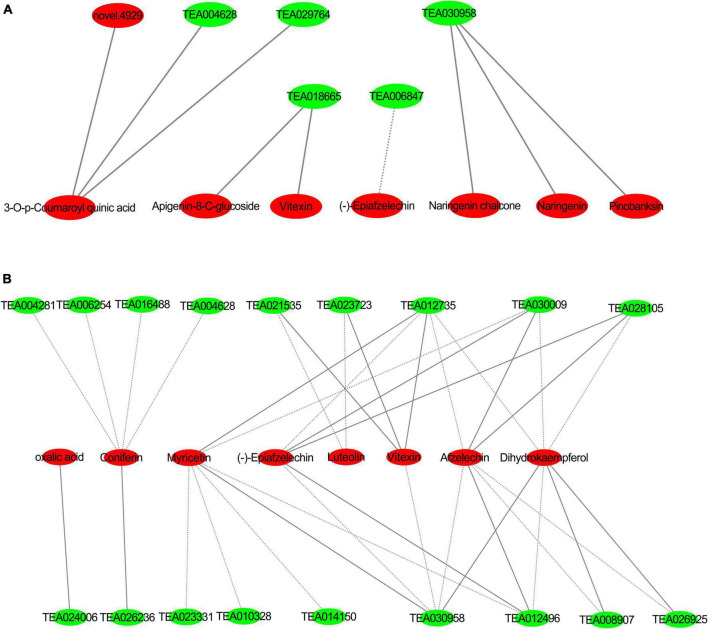
Interaction networks of differentially expressed genes and metabolites in phenylpropanoid pathway and flavonoid pathway **(A)** group JP; **(B)** group YX, red: differential metabolites, green: differentially expressed genes; solid line forward adjustment; dashed line reverse adjustment.

A correlation analysis was performed on differential genes and metabolites related to flavonoid biosynthesis and phenylpropane biosynthesis in tea plants in groups JP and YX, and the Pearson correlation coefficient R was calculated with R language, with R >0.8 being a significant correlation. The standard of Cytoscape is used to draw the interaction network diagram, and the results are as follows (Figure 12).

In group JP, caffeoyl-CoA (Tea30958), *CHS* (TEA018665), naringenin (pme0376), pinobanksin (mws0914), 4,2′,4′,6′-tetrahydroxychalcone (mws1140), apigenin8-C-pentoside (pmp000116), vitexin (mws0048) and (−)-epiafzelechin (mws1422), and other flavonoids were significantly related; among which caffeoyl-CoA positive regulated naringenin, pinobanksin, 4,2′,4′,6′-tetrahydroxychalcone dihydrokaempferol (mws1094), and other substances, and *CHS* negatively regulated the content of (−)-epicatechin and positively regulated that of vitexin and apigenin-8-C-glucoside.

In group YX, caffeoyl-CoA, *CCOAOMT* (TEA012735), *LAR* (TEA021535), 2-oxoglutarate (2OG), and Fe (II)-dependent oxygenase superfamily protein (TEA023723), anthocyanin 3-O-glucosyltransferase 2 (*UF3GT*, TEA008907) and vitexin (mws0048), afzelechin (3,5,7,4′-tetrahydroxyflavan) (pme3285), luteolin (pme0088) were significantly negatively correlated. *CCOAOMT* (TEA012735) positively regulated myricetin (mws0032) and (−)-epiafzelechin (mws1422), and reversely regulated the expression of dihydrokaempferol (mws1094). Chalcone synthase (TEA023331), flavonol synthase (TEA010328), and *HCT* (TEA014150) negatively regulates myricetin (mws0032) substances. *GH1* (TEA004281) is significantly negatively correlated with coniferin (mws0906). *CCR* (TEA026236) positively regulates the content of Coniferin (mws0906). *CAD* (TEA006254) negatively regulates the content of coniferin (mws0906).

#### Sensory evaluation of dried tea samples of groups JP and XY

The sensory evaluation and comprehensive scores of dried tea samples of group JP are shown (Table 4). Group JP has the highest total score in sensory evaluation (90.55). Moreover, the taste and leaf bottom of group JP are significantly superior to those of Tea-200 and Tea-20. The dried tea of group JP is yellow green and bright, and the liquor is yellow green and bright. It tastes mellow. The total sensory score of Tea-20 (89.45) is only next to that of Tea-1200, and its score in fragrance is the highest. Tea-20 has dense scent, yellow green and clean liquor, fat and strong appearance, green and bright color with distinctive hairs, and yellow green and fat leaf bottom. It tastes similar to Tea-200. The dried tea samples of Tea-200 (87.75) have fat and strong appearances, green and bright color with distinctive hairs, faint scent, and yellow green and fat leaf bottom.

**TABLE 4:** Results of sensory quality evaluation of dried tea samples of different ages in group JP.

Samples	Appearance (20%)		Liquor color (15%)		Fragrance (25%)		Tastes (30%)		Leave bottom (10%)		Total score
						
	Comments score		Comments score		Comments score		Comments score		Comment score		
Tea-20	Fat and strong, green and bright, tippy	89 ± 1a	Yellowish green, clean and bright	90 ± 1.32a	Dense scent	93 ± 1.8a	Strong	87 ± 1.73b	Yellowish green, fat and thick	88 ± 2.65b	89.45 ± 1.06ab
Tea-200	Fat and strong, greent, tippy	88 ± 2.65a	Yellowish green, bright	89 ± 2.18a	Faint scent	86 ± 1.73b	Strong	88 ± 0.5b	Yellowish green, fat and thick	89 ± 1b	87.75 ± 1.09b
Tea-1200	Fat and strong, green, tippy	88 ± 1.73a	Yellowish green, bright	89 ± 1.32a	Faint scent	88 ± 1.5b	Mellow, refreshing and sweet	94 ± 1a	Yellowish green, fat and thick, uniform, fresh and bright	94 ± 1.32a	90.55 ± 0.89a

Sensory evaluation and comprehensive scores of dried tea samples of Group XY are shown (Table 5). Tea-400 presents the highest total score in sensory evaluation (88.75), and it tastes significantly better than the rest of the two tea samples. Moreover, Tea-400 has higher scores in liquor color and appearance than Tea-100. It has a mellow taste, is yellow green and bright liquor, and has a fat and strong appearance and a uniform color. Its scores in fragrance and leaf bottom are significantly lower than those of Tea-100 and Tea-50. The sensory evaluation score of Tea-50 (88.15) is only next to that of Tea-400, but its scores in fragrance and leaf bottom are the highest. Tea-50 has a dense scent, is a yellow green and bright liquor, and has a fat and strong appearance a uniform color, and a uniform fat yellow green leaf bottom. However, the total score in sensory evaluation of Tea-100 is the lowest (86.6). The liquor color and taste of Tea-100 are mostly similar with those of Tea-50, and the convergence is relatively strong.

**TABLE 5:** Results of sensory quality evaluation of dried tea samples of different ages in group XY.

Samples	Appearance (20%)		Liquor color (15%)		Fragrance (25%)		Tastes (30%)		Leave bottom (10%)		Total score
						
	Comments scores		Comments scores		Comments scores		Comments scores		Comments scores		
Tea-50	Fat and strong, green, even	89 ± 1.32a	Yellowish green	86 ± 2.18b	dense scent	91 ± 1.32a	Thick	88 ± 1.8b	Yellowish green, soft and relatively bright	83 ± 2.6a	88.15 ± 1.00a
Tea-100	Fat and strong, green, relatively even	88 ± 1.73a	Yellowish green, relatively bright	87 ± 0.87b	faint scent	85 ± 0.5b	Thick	87 ± 1.5b	Yellowish green, soft and bright	86 ± 1.8a	86.60 ± 0.69b
Tea-400	Fat and strong, green, relatively even	88 ± 1.8a	Yellowish green and bright	91 ± 0.87a	faint scent	86 ± 2.65b	Mellow, fresh and sweet	92 ± 1.8a	Yellowish green, soft and relatively bright	84 ± 1.73a	88.75 ± 0.55a

To sum up, Tea-1200 and Tea-400 showed the highest sensory evaluation scores. Moreover, fragrance becomes more elegant with increase in age of tea plants, and the dense scent changed to a faint scent gradually. The liquor tastes sweeter and fresher with increase in tea plant age. Its taste changes from thick or strong to mellow, and the astringency is weakened.

## Discussion

### Differentially expressed genes of tea plants of different ages are mainly concentrated in flavonoid biosynthesis, flavonoid and flavonol biosynthesis, and phenylpropane metabolism

By network conjoint analysis of key genes and differential metabolites related with biosynthesis of flavonoids in groups JP and XY, there are six significantly correlated differential metabolites and six DEGs in group JP, as well as eight significantly correlated differential metabolites and 18 DEGs in group XY. Most flavones and key genes are positively related. This demonstrates that expressions of the structural genes related with synthesis of flavonoids are upregulated, which activates the synthesis and accumulation of flavonoids effectively (Yu et al., 2021). The coumaroyl A and trans-coumaryl coenzyme A produced by the phenylpropane pathway are precursor substances of the biosynthesis pathway of flavonoids. The phenylpropane pathway synthesizes the upstream phenylalanine deaminase (*PAL*) and cinnamate 4-hydroxylase (*C4H*). *PAL* is a multigene family that has different functions, and it participates in formation of secondary metabolites (e.g., plant flavonoids and lignins) (Mu et al., 2010; Lv et al., 2021). *PAL* locates at the inlet of the metabolic pathway of phenylalanine, and it is a rate-limiting enzyme in the pathway of shikimic acid. It catalyzes phenylalanine deamination to generate cinnamic acid (Lv, 2011). With increase in tea plant age, the expression of *PAL* increases gradually, and it reaches the highest level in Tea-400. However, expressions of flavones like vitexin and isovitexin in Tea-400 are the lowest. This indicates that *PAL* can regulate flavones. In other words, *PAL* can reverse-regulate vitexin and isovitexin. This agrees with the research conclusion of Zhang et al. (2009) that the total contents isoflavone in soybean leaves, contents of three aglycones, and relative expression of *PAL* in leaves have a synergistic growth and reduction trends. As the second enzyme in the phenylpyruvic acid pathway, *C4H* catalyzes the hydroxylation of trans-cinnamic acid and generates 4-cumaric acid. Transcriptional abundance may influence the synthesis pathway of lignins and flavones in plants (Jin et al., 2010). In this study, the expression level of *C4H* in the Tea-400 of group YX is significantly higher than those of Tea-100 and Tea-50. In group JP, the expression level of *C4H* in Tea-200 is significantly higher than those of Tea-20 and Tea-1200. *4CL* and *CHS* are at the downstream of the biosynthesis pathway of flavonoids. *4CL*, *CHS*, and CHI catalyze the formation of naringenin (Luo et al., 2009). Expressions of *CHS* and *4CL* in Tea-100 and Tea-200 are the highest. Expressions of differential metabolites such as naringenin, aromadendrin, and chalcone in Tea-100 and Tea-200 are significantly higher than those in the other tea plant samples. Therefore, *4CL* and *CHS* can positively regulate the biosynthesis of substances like naringenin, aromadendrin, and chalcone. Therefore, we speculate that the differential expression of several enzyme genes such as *PAL*, *C4H*, and *CHS* may be the main reasons for the difference in metabolites of fresh leaves of different tea plant ages.

### Younger tea plants have more vigorous metabolism than older tea plants

According to the analysis of transcriptional sequencing of groups JP and XY, a total of 20,788 and 8,971 DEGs were screened, respectively. For group JP, there are 10,108 upregulated genes and 10,680 downregulated genes. In group XY, there are 4,163 upregulated genes and 4,804 downregulated genes. The number of downregulated genes in both groups JP and XY is higher than that of upregulated genes. In other words, the quantity of DEGs is positively related with age of tea plants. The expression levels of differential genes between the younger and older tea plants are significantly different. Therefore, the younger tea plants have more vigorous metabolism. This agrees well with the conclusion of Liu et al. (2022) on the metabolism and transcriptional results of ginkgo trees of different ages: the metabolism of younger leaves is more vigorous than that of old leaves, and the monthly genetic expressions of young trees generally have significant differences, while age differences lead to more obvious differences in flavones and terpenes of ginkgo trees.

### Difference in metabolites of fresh leaves of tea plants of different ages is the reason for difference in their dried tea quality

Tea is a kind of healthy beverage, and it is highly appreciated by consumers for its rich taste (Xu et al., 2021). Secondary metabolites like tea polyphenol (TP), caffeine, and amino acids are closely related with tastes and functions of tea (Huang et al., 2018). The TP in fresh leaves of tea plants is a major component of secondary metabolites of tea, and it influences tea quality (Tang et al., 2015). TP is mainly composed of catechinic acids, flavones and flavonols, anthocyanin and leucoanthocyanidin, phenolic acid, and depside (Yang et al., 2013). Fresh leaves of high quality are the vital foundation of forming an excellent quality of tea. Some study pointed out that the material basis of the finished quality of wild tea is better than that of terraced tea (Bai et al., 2018). However, relevant studies are not comprehensive. In this study, metabolite differences among fresh leaves from tea plants of different ages and relevant regulation mechanism were analyzed by metabolomics and transcriptomics. According to the metabolomics sequencing of groups JP and XY, differential metabolites between older tea plants and younger tea plants are mainly flavonoids, polyphenols (phenolic acids and tannin), and alkaloids. Moreover, the quantity of downregulated flavonoids in older tea plants is higher than that of upregulated ones, which might be related with senescence of old tea plants. Polyphenols are mainly related with biosynthesis of phenylpropane and flavonoids. Specifically, flavone substances exist widely in various organs of plants. They not only participate in formation of flowers, fruits, and seed colors but also protect plants from ultraviolet damage and prevent the invasion of pathogenic microorganism (Chen et al., 2011). Flavones in fresh leaves of tea plants are gentle and astringent, and can increase the bitter taste of caffeine. Flavones are one of the factors that influence liquor color and taste quality, resulting in bitter taste (Zeng, 2015). In this study, the expressions of flavones of Tea-1200 are significantly lower than those of Tea-20 and Tea-200. According to the sensory evaluation of dried tea samples of groups JP and XY, the liquor of wild tea tastes sweeter and fresher but less bitter, while liquor of the younger tea is more bitter and lasting. Zhang (2018) found that flavones are one of the influencing factors of the liquor color of tea. Wild tea has lower content of flavones than terraced tea, and it tastes fresher and less bitter. This is consistent with the conclusions of this study. Amino acids are major refreshing substance in tea, and they account for about 3% of total dry substances (Hu et al., 2017). In this study, the expressions of amino acids (e.g., DL-proline, xanthine, L-glutamine, and L-proline) and their derivatives were analyzed, and it was found that contents of amino acids decrease with increase in tea plant age. This indicates that contents of amino acids in younger tea samples are higher than that in older tea samples. This is consistent with the research conclusion of Wang (2020) when studying the formation mechanism of quality differences of Wuyi rock tea samples of different stages: contents of amino acids in fresh leaves decrease gradually with increase in tea plant age.

## Data availability statement

The datasets presented in this study can be found in online repositories. The names of the repository/repositories and accession number(s) can be found below: https://www.ncbi.nlm.nih.gov/bioproject/PRJNA869892.

## Author contributions

SJ conceived the study, organized, and implemented the study. SJ and YZZ conducted the experiments and evaluated the result together with MYL. MYL and YZZ wrote the manuscript. SJ revised the manuscript. ZYZ and YHZ provides experimental materials and investigated the relevant information about the materials. QQR participated in sampling and data analysis. All authors discussed the results and content and contributed to the article and approved the submitted version.

## Footnotes

1
http://tpdbtmp.shengxin.ren:81/analyses_search_locus.html
