# Supplementary material for: Differences in transcriptomic and metabolomic analyses of metabolites of shoots on tea plants of different ages and relevant regulatory network
Source: Front Plant Sci. 2023 Mar 2;13:910895. doi: 10.3389/fpls.2022.910895 (PMC10019279; doi:10.3389/fpls.2022.910895)
Supplement: Supplementary file 1 [file Data_Sheet_1.docx]

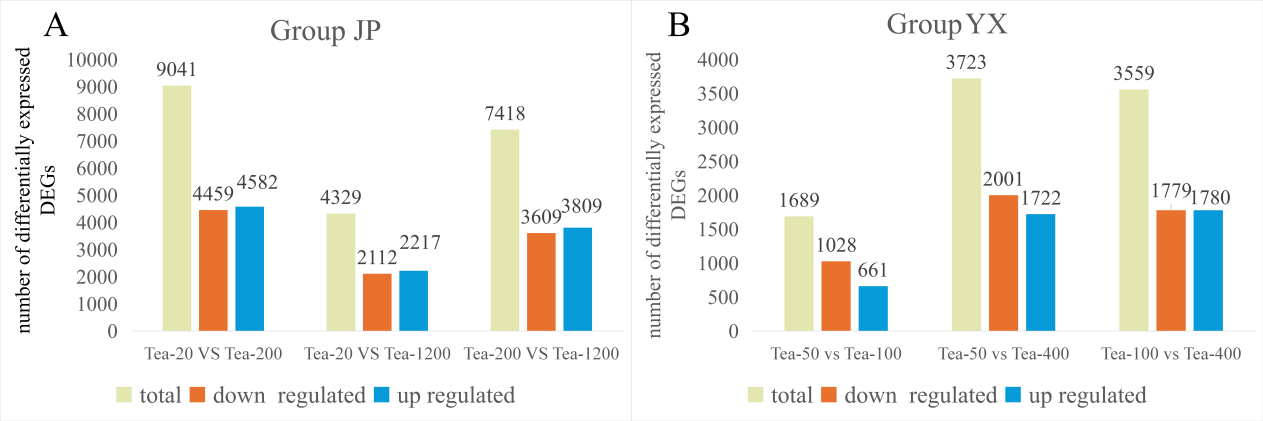


**FIGURE 1 |** Histograms of DEGs of Group JP and Group YX .Group JP(A),B: Group YX(B).


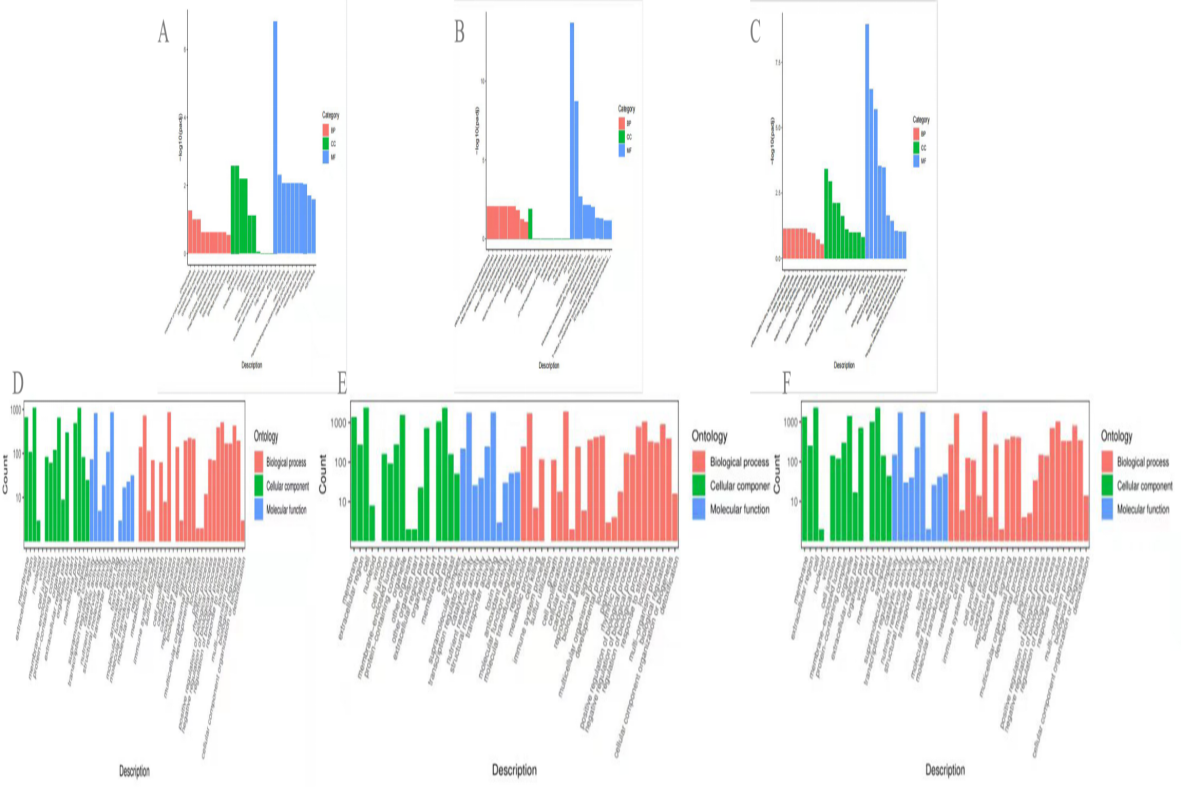


**Figure 2** | GO enrichment analysis of DEGs in Group JP and Group YX. Tea-20 VS Tea-200(A),Tea-20 VS Tea-1200(B), Tea-200 VS Tea-1200(C),Tea-50 VS Tea-100(D),Tea-50 VS Tea-400(E),Tea-100 VS Tea-400(F).


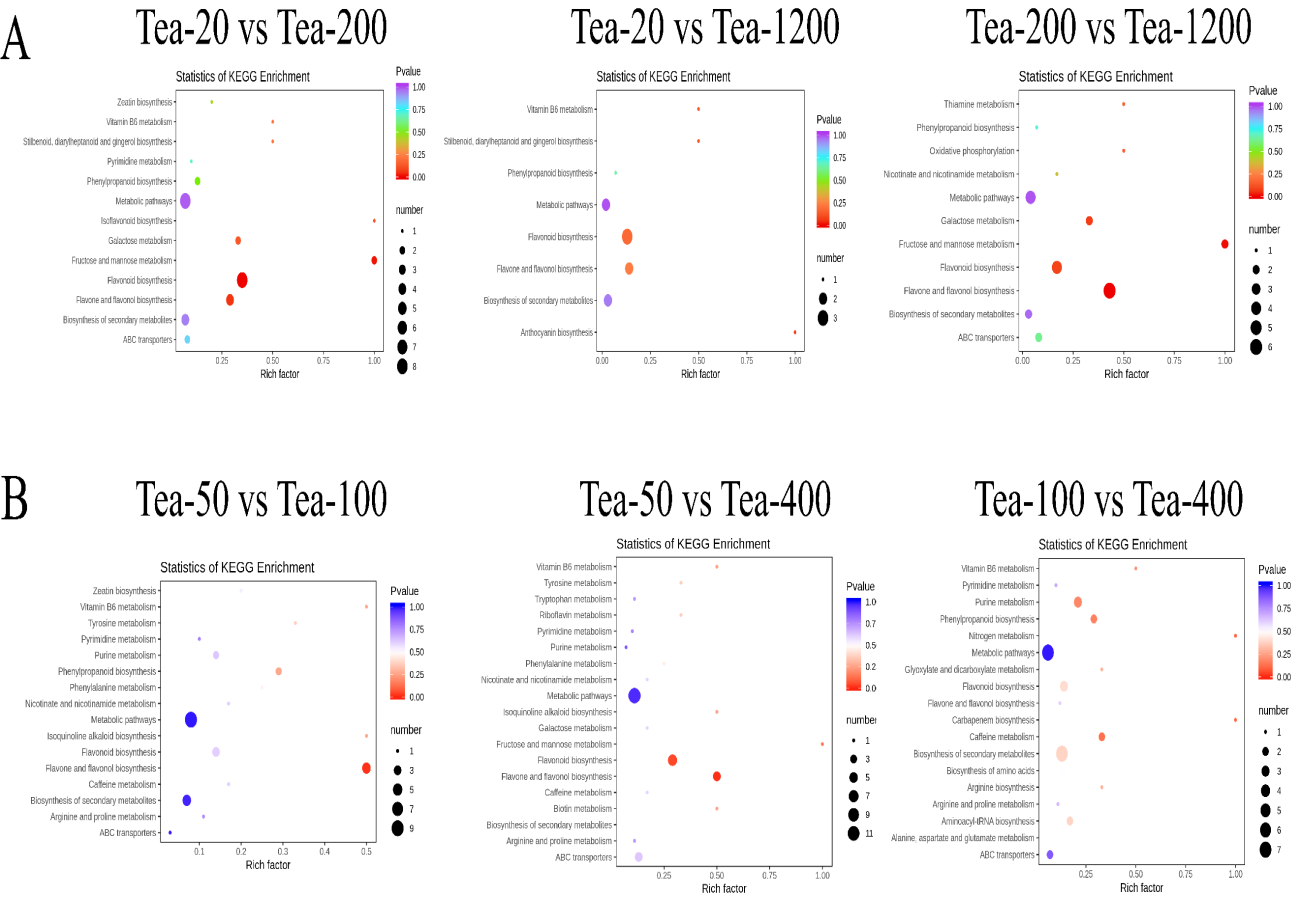


**Figure 3** | KEGG pathway enrichment analysis of DEGs in Group JP and Group XY. Group JP(A),Group YX(B).


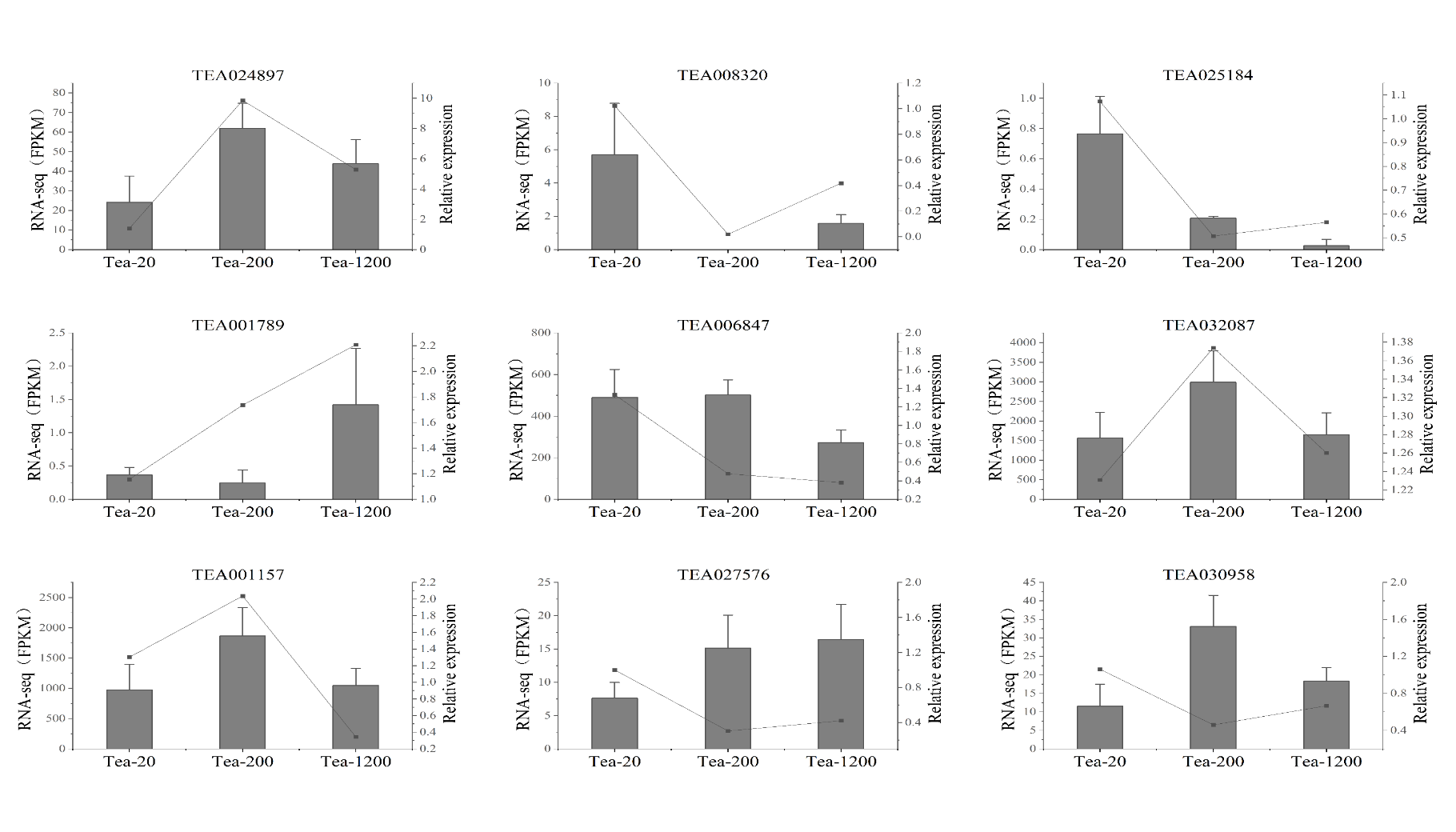


**Figure 4 |** qRT-PCR verification of transcriptomic sequencing. FPKM of transcriptome data (grey columns) and relative expressions of qRT-PCR test (black points) are represented.


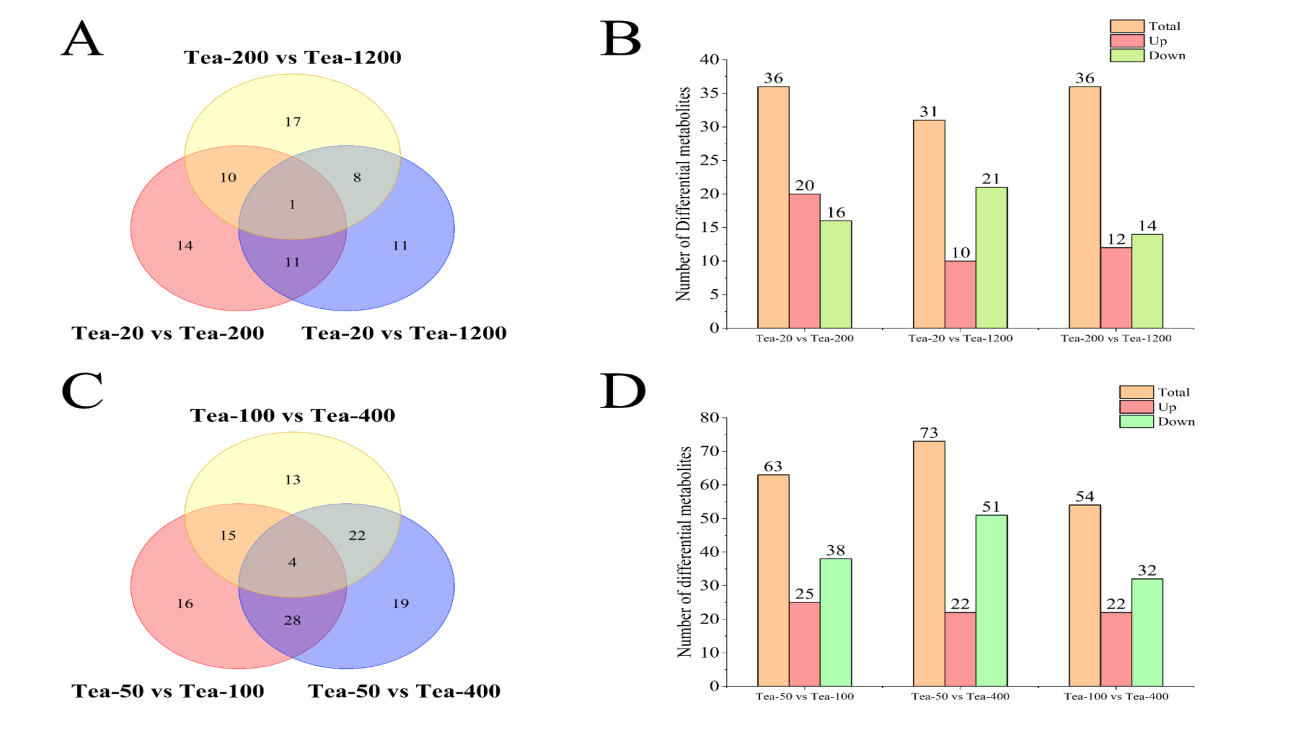


**Figure 5 |** Venn diagram and histogram of differential metabolites in Group JP and Group YX. Group JP(A B), Group YX(C D).


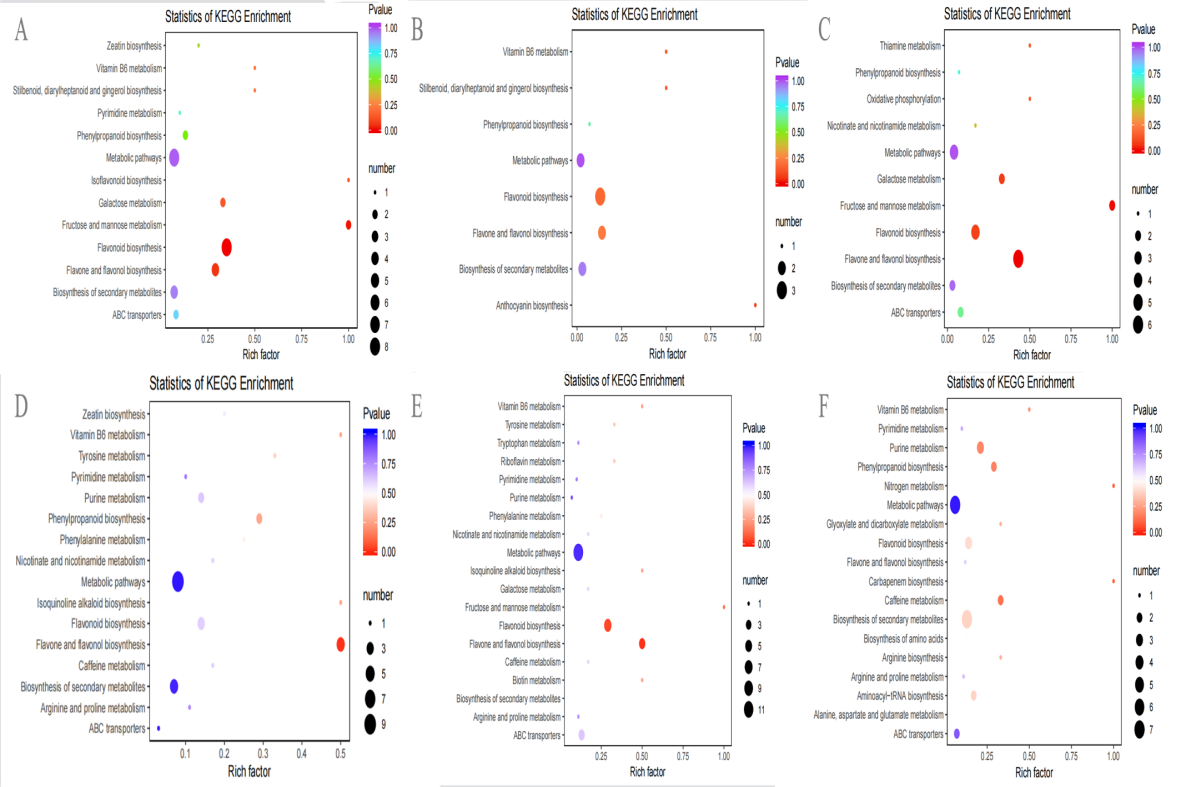


**Figure 6 |** KEGG pathway enrichment analysis of differential metabolites of Group JP and Group YX. Tea-20 VS Tea-200(A),Tea-20 VS Tea-1200(B), Tea-200 VS Tea-1200(C), Tea-50 VS Tea-100(D),Tea-50 VS Tea-400(E), Tea-100 VS Tea-400(F).


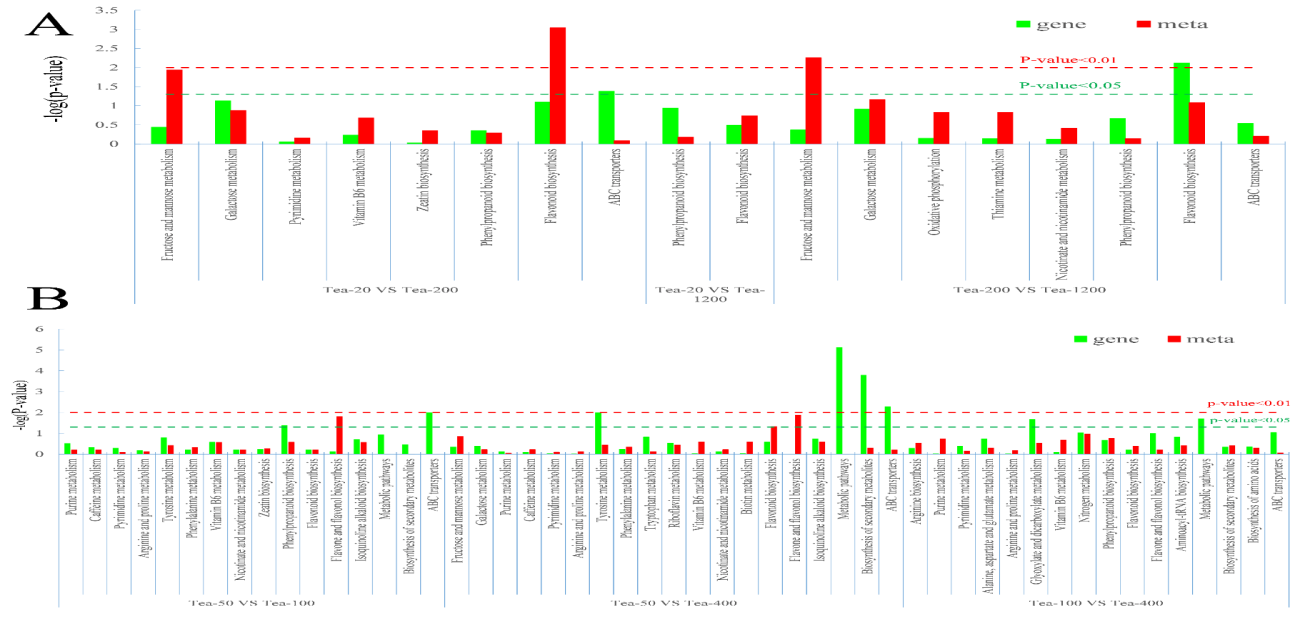


**Figure 7 |** Degree of relevant pathway enrichment of differential metabolites and DEGs.Group JP(A),Group YX(B).


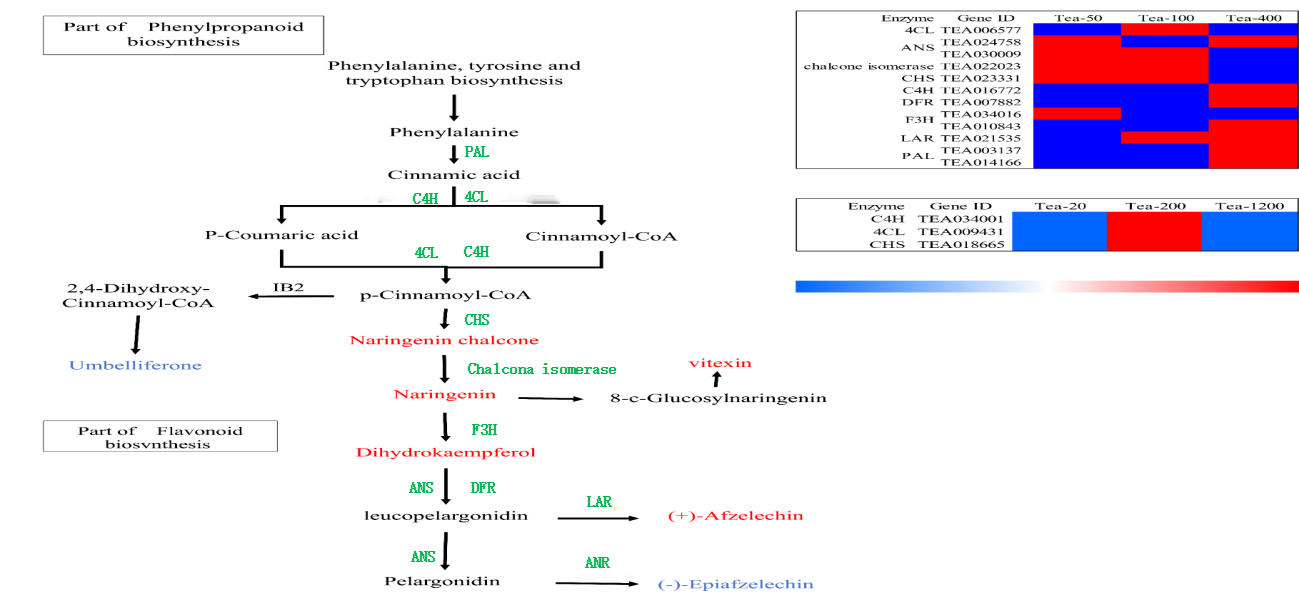


**Figure 8 |** Synthesis of flavonoids in shoot of different tea trees and relevant gene expression heat mapCorrelations of differential metabolites and DEGs on the biosynthesis pathway of flavonoids were analyzed.


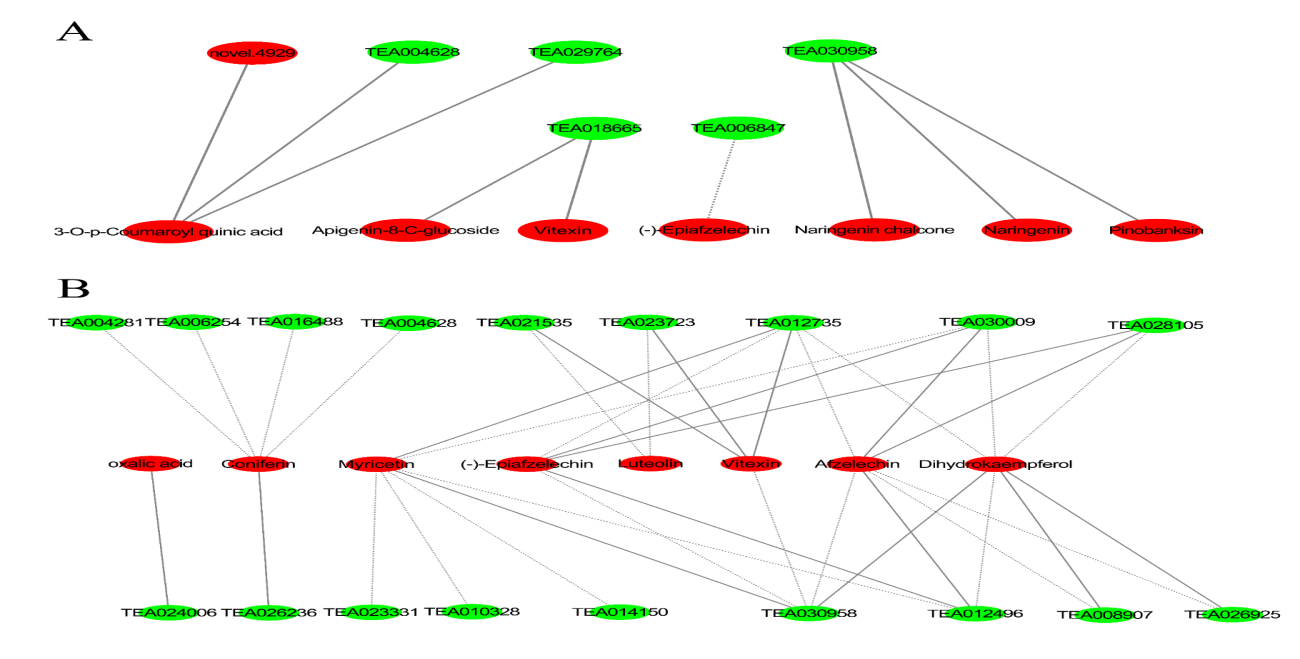


**Figure 9 |** Interaction network of DEGs and differential metabolites in the phenylpropane pathway and flavonoids pathway. Group JP(A),Group YX(B),differential metabolites(Red), DEGs(green), positive regulation(solid line),reverse regulation(dotted line)
